# Prognostic impact of tumor budding in rectal cancer after neoadjuvant therapy: a systematic review and meta-analysis

**DOI:** 10.1186/s13643-023-02441-9

**Published:** 2024-01-09

**Authors:** Jinghui Li, Yongli Ma, Liang Wen, Guosheng Zhang, Chengzhi Huang, Junjiang Wang, Xueqing Yao

**Affiliations:** 1 Gannan Medical University, Ganzhou, China; 2 Ganzhou Hospital of Guangdong Provincial People’s Hospital, Ganzhou Municipal Hospital, Ganzhou, China; 3 Department of Gastrointestinal Surgery, Department of General Surgery, Guangdong Provincial People’s Hospital(Guangdong Academy of Medical Sciences), Southern Medical University, Guangzhou, 510080 China

**Keywords:** Tumor budding, Rectal cancer, Neoadjuvant therapy, Prognosis, Pathological features

## Abstract

**Background:**

Tumor budding (TB) is a negative prognostic factor in colorectal cancer; however, its prognostic impact following neoadjuvant therapy for patients with rectal cancer remains unclear. This study aims to assess the prognostic impact of TB and the correlation between TB and other pathological features in patients with rectal cancer after neoadjuvant therapy.

**Methods:**

A comprehensive search of PubMed, Embase, Cochrane, Scopus, CNKI, Wanfang, and ClinicalKey databases was conducted for studies on the prognosis of TB in rectal cancer after neoadjuvant therapy from the inception of the databases to January 2023, and the final literature included was determined using predefined criteria. Quality assessment of the studies included, extraction of general and prognostic information from them, and meta-analyses were carried out progressively.

**Results:**

A total of 11 studies were included, and the results of the meta-analysis showed that high-grade tumor budding (TB-1) increased the risk of poor 5-year disease-free survival (HR = 1.75, 95% CI 1.38–2.22, *P* < 0.00001), 5-year overall survival (HR = 1.77, 95% CI 1.21–2.59, *P* = 0.003), local recurrence (OR = 4.15, 95% CI 1.47–11.75, *P* = 0.007), and distant metastasis (OR = 5.36, 95% CI 2.51–11.44, *P* < 0.0001) in patients with rectal cancer after neoadjuvant therapy. TB-1 was significantly associated with poor differentiation and lymphatic, perineural, and venous invasion.

**Conclusion:**

Tumor budding is significantly correlated with unfavorable prognosis and poor pathological characteristics following neoadjuvant therapy for rectal cancer. We anticipate more high-quality, prospective studies in the future to confirm our findings.

**Systematic review registration:**

PROSPERO CRD42022377564.

**Supplementary Information:**

The online version contains supplementary material available at 10.1186/s13643-023-02441-9.

## Background

Neoadjuvant therapy is widely used in the treatment of locally advanced rectal cancer because it markedly reduces the tumor stage and improves patients’ chances of undergoing radical surgery [1, 2]. With the development of neoadjuvant therapies such as combined oxaliplatin neoadjuvant therapy, short-course neoadjuvant chemoradiotherapy (nCRT), neoadjuvant immunotherapy, total neoadjuvant chemoradiotherapy (TNT), contact X-ray brachytherapy, and radiotherapy dose escalation, the tumor control and survival benefits of patients with rectal cancer have been clearly enhanced [2–8]. Currently, however, the treatment of rectal cancer is mainly based on the tumor-node-metastasis (TNM) staging system, and it has been found that not all patients with rectal cancer have good outcomes from the classic model of “neoadjuvant therapy-radical resection-postoperative adjuvant therapy” prompting researchers to explore better markers to assist in identifying patients with rectal cancer suitable for this treatment model to achieve precise treatment [9, 10].

Tumor budding (TB) is a negative pathological marker for colorectal cancer and other tumors and is defined by the International Tumor Budding Consensus Conference (ITBCC) as the presence of a single tumor cell or a cluster of more than four tumor cells. Depending on the location of the source of the pathological specimens, they can be classified as follows: peritumoral budding (PTB), which can only be detected in surgical resection specimens, and intratumoral budding (ITB), which can be detected in biopsies and surgical specimens [11–13]. TB has been demonstrated to be associated with pT1 colorectal cancer lymph node metastasis, and meta-analyses and original studies have found that high-grade TB is associated with poor clinical outcomes in patients with colorectal cancer [14–17]. However, many studies and meta-analyses analyzed colon and rectal cancers together as a single population and have not controlled for neoadjuvant therapy which has been shown to be an important prognostic factor for rectal cancer. In 2012, Du et al. found that the morphology of TB was altered after neoadjuvant therapy for rectal cancer and that cancer cells in the gland showed a “false budding” pattern after radiation therapy subsided, which may make the assessment of TB more difficult. This study also found that high-grade tumor budding was an independent poor prognostic factor for 5-year overall survival (OS) but did not correlate with the degree of response to neoadjuvant therapy [18]. However, subsequent studies have consistently shown that positive or high-grade TB, whether PTB or ITB, is strongly associated with a poorer response to neoadjuvant therapy for rectal cancer [19–24]. At the same time, researchers have offered different insights into aspects of the prognostic and predictive value of TB in patients with rectal cancer after neoadjuvant therapy [13, 18, 20–28]. Recently, it has been shown that TB and lymphatic, perineural, and venous invasion are all important prognostic factors affecting the 5-year disease-free survival (DFS) and OS of patients undergoing radical surgery for rectal cancer after neoadjuvant therapy [29].

Therefore, the aim of our study was to pool published studies to (1) assess the impact of TB on the prognosis of rectal cancer after neoadjuvant therapy and (2) assess its correlation with other pathological features, to determine if it could aid in clinical decision-making.

## Methods

### Search and screening of literature

PubMed, Embase, Cochrane, Scopus, CNKI, Wanfang, and ClinicalKey databases were searched for literature related to both TB and neoadjuvant therapy for rectal cancer. The search period was from the establishment of the database to January 2023, and the language was not limited. To make the search as complete as possible, the following search terms were selected: tumo(u)r budding, budding of tumo(u)r, budding, rectal neoplasm, rectal tumor, cancer of rectum, rectum cancer, cancer of the rectum, rectal cancer, prognosis, overall survival, OS, disease-free survival, DFS, local recurrence, LR, distant metastasis, and DM (search strategy in Additional file 1). The search results were screened independently by two researchers to identify studies that matched the criteria. This systematic review and meta-analysis was conducted under the PRISMA2020 guidelines and has been registered in PROSPERO (registration number: CRD 42022377564).

### Inclusion and exclusion criteria

#### Inclusion criteria

(1) The study population included patients who underwent radical surgical resection of rectal adenocarcinoma after neoadjuvant therapy. (2) Studies with comparisons between high-grade or positive (TB-1) and low-grade or negative (TB-0) tumor budding groups and providing survival analysis data. (3) The full text was fully accessible, and relevant data could be extracted. (4) The study type was randomized controlled, case–control, and cohort studies.

#### Exclusion criteria

The exclusion criteria are (1) studies without follow-up and (2) studies with no separate analysis of comparative survival between the two groups with rectal cancer after neoadjuvant treatment.

### Bias and quality assessment

If the final included studies were cohort and case–control studies, we assessed the bias and quality of the included studies using a Modified Newcastle Scale (NOS). The rating of NOS is nine stars in total: low-quality research, one to three stars; medium-quality research, four to six stars; and high-quality research, seven to nine stars. Randomized controlled trials were assessed using the Cochrane Evaluation Scale.

### Data extraction

Information on the basic characteristics of the included studies, including first author, year of publication, country, type of study, grouping criteria, type of budding, number of cases in both groups, age, sex, tumor stage, distance of the tumor from the anal verge, neoadjuvant regimen, interval between neoadjuvant treatment and surgery, mode of surgery, postoperative adjuvant regimen, and follow-up period, was extracted independently by two reviewers. Pathological data included specimen source, staining method, degree of differentiation, and lymphatic, perineural, and venous invasion. Outcomes included OS, DFS, cancer-specific survival (CSS), local recurrence (LR), and distant metastasis (DM). Survival analysis data were extracted from the original multivariate regression analysis for hazard ratios (HR) and 95% confidence intervals (CI) or from extracted Kaplan–Meier curve data using the Engagement digitizer software, with subsequent statistical transformation using data tables developed by Tierney et al.

### Statistical analysis

A meta-analysis of outcome indicators for more than three included studies was performed using the Review Manager 5.4 and stata17 software. The 5-year DFS and OS data were pooled using HRs and 95% CIs; LR, DM, degree of differentiation, lymphatic invasion, perineural invasion, and venous invasion data were pooled using the odds ratios (OR) and 95% CIs. Notably, Trotsyuk et al. compared two pathological staining methods, and to minimize heterogeneity among the included studies, we selected data related to HE staining for the meta-analysis [23]. A random-effects model was used to conduct a meta-analysis of all outcomes and pathological characteristics. Differences in the meta-analysis results were considered statistically significant if the combined overall effect was *P* < 0.05. Funnel plots and Egger tests were performed on the 5-year DFS data to assess publication bias, and sensitivity analyses were performed to assess the robustness of the pooled results. In this study, subgroup analysis was conducted only for the outcome indicator of 5-year DFS; however, other outcome indicators were not available for subgroup analysis.

## Results

### Search and filter results

**Fig. 1:**
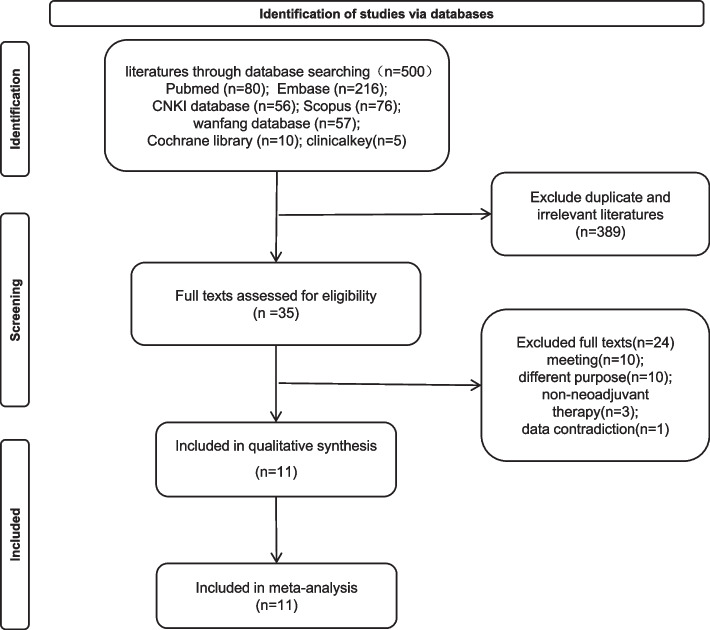
Flow chart of literature screening

A total of 500 papers were retrieved, and titles and abstracts were browsed using the Endnote software (version 20.0) to exclude duplicate publications and those that failed to match the inclusion criteria. Thirty-five papers were initially screened, and the full text was obtained and browsed, resulting in the inclusion of 11 studies with a total of 2178 cases, the details of which are shown in Fig. 1. All 11 studies were retrospective, of which 1 was a case–control study and the remaining 10 were cohort studies. They were considered medium- to high-quality studies because the NOS assessments were all above 5 stars (Additional file 2). Patients with rectal cancer were treated with neoadjuvant radiotherapy in 2 of the included studies, some patients received TNT in 1 study, 3 studies involved neoadjuvant or adjuvant chemotherapy with oxaliplatin, and 4 studies did not provide information on adjuvant chemotherapy. Specimens assessed for TB were derived from pretreatment biopsies in three studies and from surgically resected specimens in eight. Detailed information is shown in Table 1, 2, 3, and 4.

**Table 1:** Information on the basic characteristics of the included studies

Authors	Year	Country	Number of cases		Age*		Gender (male and proportion,%)		Distance from anal edge (cm)^a^		Follow-up months^b^	Type of study^c^	Stars of NOS
			TB-1	TB-0	TB-1	TB-0	TB-1	TB-0	TB-1	TB-0			
C. Du [18]	2012	China	36	48	< 60: ≥ 60 = 25: 11	< 60: ≥ 60 = 25: 23	18 (50)	31 (64.6)	≤ 5: ≥ 5 = 13: 23	≤ 5: ≥ 5 = 15: 34	71	Case–control	5
M. Huebner [25]	2012	America	24	210	60.0 (12.49)		160 (67.5)		NA		42	Cohort	7
A. C. Rogers [20]	2013	Ireland	18	71	59.3 (8.6)	62.3 (12.8)	12 (67)	45 (63)	NA		49	Cohort	7
J. W. Huh [28]	2016	Korea	44	165	56 (27–81)		136 (65)		≤ 10		44	Cohort	6
T. Jäger [21]	2018	Switzerland	81	47	64 (10)	63 (10)	57 (66)	30 (34)	NA		84^a^	Cohort	7
M. Swets [24]	2018	Holland	99	199	59.8 (9.7)		136 (61.5)		≤ 10: > 10 = 130:85		64.8	Cohort	7
A. Demir [26]	2019	Turkey	46	29	56 (19–77)		31 (68)	20 (69)	P: 21; M: 19; D: 44	P: 7; M: 11; D: 11	35	Cohort	6
J. W. Huh [22]	2019	Korea	43	452	56 (24–80)		437 (68.4)		4.0	4.0	56.7	Cohort	6
I. Trotsyuk [23]	2019	Germany	38	65	67.1 (10.8)	62.5 (9.2)	25 (65.8)	48 (73.8)	NA		54.7^d^	Cohort	6
L. Farchoukh [13]	2021	America	26	91	65 (20)	60 (17)	17 (65)	52 (57)	M: 10 D: 16	M: 43 D: 48	29	Cohort	7
J. K. Shin [27]	2021	Korea	209	209	< 65: ≥ 65 = 154: 55	< 65: ≥ 65 = 146: 63	147 (70.3)	146 (69.9)	NA		NA	Cohort	7

*NA* not applicable

^*^Age: mean and SD; median and range

^a^Follow-up month: mean month (day); other was a median month

^b^Median distance from the anal edge: *P* proxima, *M* middle or mid-rectum, *D* distal or distal rectum

^c^Type of study: all included studies were retrospective study

**Table 2:** Information on the basic characteristics of the included studies

Authors	Year	TB-1	Type of budding	Stage of tumor		Neoadjuvant therapy	Interval (weeks)	Pathology source	Recognition methods
				TB-1	TB-0				
C. Du	2012	≥ 10	PTB	ypT1–2:ypT3–4 = 13:23, ypN0:ypN + = 16:20	ypT1–2:ypT3–4 = 20:28, ypN0:ypN + = 34:14	nRT	2–3	Postoperative specimens	H&E/IHC
M. Huebner	2012	≥ 10	PTB	yp0 = 39; yp1 = 14; yp2 = 25; yp3 = 144; yp4 = 15		CRT	6–8	Postoperative specimens	H&E
A. C. Rogers	2013	≥ 1	ITB	ypT0–1 = 0; ypT2 = 4; ypT3 = 11; ypT4 = 3	ypT0 = 10; ypT1 = 6; ypT2 = 16; ypT3 = 33; ypT4 = 6	CRT	6–8	Pretreatment biopsies	H&E
J. W. Huh	2016	NA	NA	II 26; III 183		CRT	6–8	Pretreatment biopsies	IHC
T. Jäger	2018	≥ 5	PTB	pT0 = 0; pT1 = 2; pT2 = 17; pT3 = 57; pT4 = 5, pN − = 52; pN + = 29	pT0 = 16; pT1 = 5; pT2 = 19; pT3 = 7; pT4 = 0, pN − = 40; pN + = 7	CRT	90 days	Postoperative specimens	H&E
M. Swets	2018	≥ 5	PTB	II = 29; III = 192		nRT	NA	Postoperative specimens	H&E
A. Demir	2019	≥ 10	PTB	NA		CRT	8–12	Postoperative specimens	H&E
J. W. Huh	2019	NA	NA	pT0 = 154; pT1 = 29; pT2 = 165; pT3 = 278; pT4 = 13, pN − = 450; pN + = 189		CRT	6–8	Postoperative specimens	NA
I. Trotsyuk	2019	≥ 5	PTB&ITB	ypT0–1 = 0; ypT2 = 6; ypT3 = 26; ypT4 = 6, ypN − = 12; ypN + = 26	ypT0–1 = 10; ypT2 = 16; ypT3 = 25; ypT4 = 4, ypN − = 40; ypN + = 25	CRT	4–6	Postoperative specimens	H&E/IHC
L. Farchoukh	2021	≥ 2	ITB	0–I = 49, II–III = 68		CRT/TNT	NA	Pretreatment biopsies	H&E
J. K. Shin	2021	≥ 5	PTB	ypTI = 26; ypTII = 72; ypTIII = 111	ypTI = 24; ypTII = 73; ypTIII = 112	CRT	6–8	Postoperative specimens	H&E

*NA* not applicable, *nRT* neoadjuvant radiotherapy, *CRT* neoadjuvant chemoradiotherapy, *TNT* total neoadjuvant chemoradiotherapy

**Table 3:** Tumor outcome information

Authors	Year	Outcome	TB-1	TB-0	*P*-value	HR	HR_LI	HR_UI
C. Du	2012	5-year DFS	55.6%	87.5%	< 0.001	1.45	0.13	15.71
M. Huebner	2012	5-year CSS	73.8%	88.4%	–	6.73	1.56	28.96
A. C. Rogers	2013	5-year DFS	33.3%	77.5%	≤ 0.001	3.49	0.73	16.60
		5-year CSS	61.1%	87.3%	0.021	5.79	0.44	76.62
		LR	33%	10%	0.012	–	–	–
		DR*	44%	15%	0.007	–	–	–
		5-year CSD	39%	13%	0.01	3.51	1.03	11.93
J. W. Huh	2016	5-year DFS	–	–	–	1.109	0.593	2.073
		LR	–	–	–	2.040	0.766	5.429
T. Jäger	2018	5-year RFS	71%	90%	0.02	3.44	1.23	9.63
		5-year OS	80%	90%	0.09	–	–	–
		LR	7%	0%	0.27	–	–	–
		DR	12%	2%	0.03	–	–	–
M. Swets	2018	5-year DFS	–	–	–	1.54	1.00	2.37
		5-year OS	–	–	–	1.54	1.09	3.03
		DR	–	–	–	1.60	1.00	2.57
A. Demir	2019	1-year DFS	86%	93%	0.01	–	–	–
		3-year DFS	24%	61%	0.01	–	–	–
J. W. Huh	2019	5-year OS	–	–	–	1.48	0.535	4.094
I. Trotsyuk	2019	5-year DFS^a^	39%	75%	< 0.001	2.34	1.14	4.79
		5-year OS^a^	53%	84%	0.001	2.72	1.15	6.44
		5-year DFS^b^	44%	87%	< 0.001	4.59	1.79	11.72
		5-year OS^b^	59%	92%	< 0.001	5.19	1.62	16.61
L. Farchoukh	2021	5-year DFS	39%	87%	0.001	3.35	1.25	8.99
		LR	4%	2%	–	–	–	–
		DR	31%	7%	–	–	–	–
J. K. Shin	2021	5-year DFS	65.4%	80.5%	< 0.001	1.665	1.108	2.504
		5-year OS	82.1%	94.7%	< 0.001	2.102	1.111	9.979

Methods of pathological staining of the specimen

*NA* not applicable

^*^Distant recurrence (DR): also known as distant metastasis (DM), refers to the tumor involving the peritoneum, liver, lung sites, and other distant organs

^a^Hematoxylin and eosin (H&E) staining

^b^Immunohistochemical (IHC) staining

**Table 4:** Information on preoperative radiotherapy and postoperative chemotherapy

Authors	Year	Preoperative chemoradiotherapy regimen	Way of surgery	Postoperative chemotherapy
C. Du	2012	3000 cGy in 10 fractions delivered within 2 weeks, with a biologic equivalent dose of 36 Gy	APR, LAR (TME)	8 to 12 cycles of postoperative chemotherapy based on 5 FU or capecitabine
M. Huebner	2012	Combination of irradiation and 5-fluorouracil (5-FU) chemotherapy	APR, AR, LAR	Postoperative 5-FU chemotherapy
A. C. Rogers	2013	45–50.4 Gy in 25–28 fractions of 1.8 Gy delivered over 6 weeks. 5-Fluorouracil was given concomitantly by protocol	APR, ultra-LAR with coloanal anastomosis, LAR, AR, pelvic exenteration, or a Hartmann’s procedure	NA
J. W. Huh	2016	40.4 to 50.4 Gy and concomitant chemotherapy based on a 5-fluorouracil or capecitabine regimen	Radical resection	A 5-fluorouracil-based regimen (*n* = 160, 82.1%), a capecitabine (*n* = 12, 6.1%), an oxaliplatin-based regimen (*n* = 13, 6.7%), and other regimens (*n* = 10, 5.1%)
T. Jäger	2018	Oral capecitabine or intravenously administered 5-fluoruracil during 6 weeks of radiotherapy. For the patients (52%), oxaliplatin was used as an adjunct to the concomitant chemotherapy	LAR, APR	Of the 128 patients, 47.9% (58 of 121) received fluoropyrimidine (5-FU/leucovorin or capecitabine) in all patients except one. Sixty-four percent (37 of 58 patients) additionally received oxaliplatin
M. Swets	2018	Neoadjuvant short-course radiotherapy (5 × 5 Gy)	TME	104 patients were randomized assigned to adjuvant chemotherapy and 117 patients to observation
A. Demir	2019	45 Gy/28 days. Capecitabine 825 mg/m^2^/day or 5-fluorouracil 200 mg/m^2^ D1–5 weekly was administered	AR, LAR, ultra-LAR, miles, total colectomy	NA
J. W. Huh	2019	Preoperative 5-fluorouracil-based chemotherapy and pelvic radiation (4040–5040 cGy)	LAR, APR, Hartmann’s procedure	425 (92.2%) received adjuvant chemotherapy
I. Trotsyuk	2019	Eighty patients: 50.4 Gy applied in 5 weekly fractions of 1.8 Gy using 18-MeV photons and received a continuous infusion of 225 mg 5-FU per day and square meter of body surface for the duration of radiotherapy. Most of the remaining 44 patients received only slightly variant chemotherapy along with hyperfractionated radiation	APR, LAR	Adjuvant chemotherapy was received in 71 (57.3%) cases, while in 32 (25.8%) cases, the tumor board decided against adjuvant therapy. For 21 (16.9%) patients, information on adjuvant therapy was not available
L. Farchoukh	2021	nCRT: (50.4 Gy) and concurrent 5-fluorouracil chemotherapy. TNT: 5-fluorouracil, leucovorin, and oxaliplatin (FOLFOX) followed by preoperative radiotherapy with concurrent 5-fluorouracil	Surgical resection	NA
J. K. Shin	2021	4500–5400 cGy in 5–6 weeks with synchronous 5-fluorouracil-based chemotherapy	Radical resection	NA

*APR* abdominal perineal resection, *AR* anterior resection, *LAR* low anterior resection, *TME* total mesorectal excision

### Results of meta-analysis

#### Oncology outcomes

##### DFS and OS

**Fig. 2:**
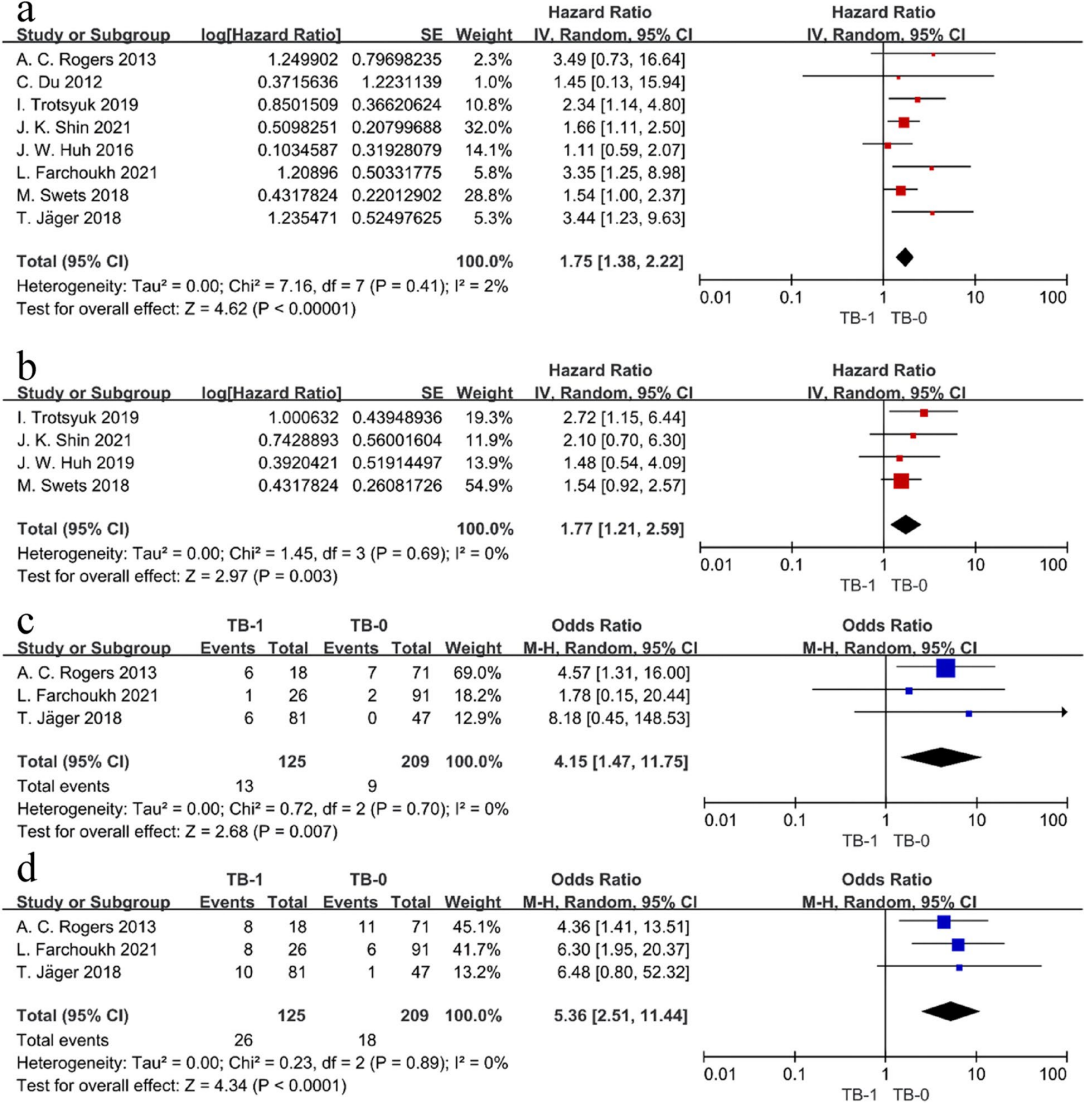
Forest plot comparing oncology outcomes between the TB-1 versus TB-0 groups. **a** 5-year DFS. **b** 5-year OS. **c** LR. **d** DM

**Fig. 3:**
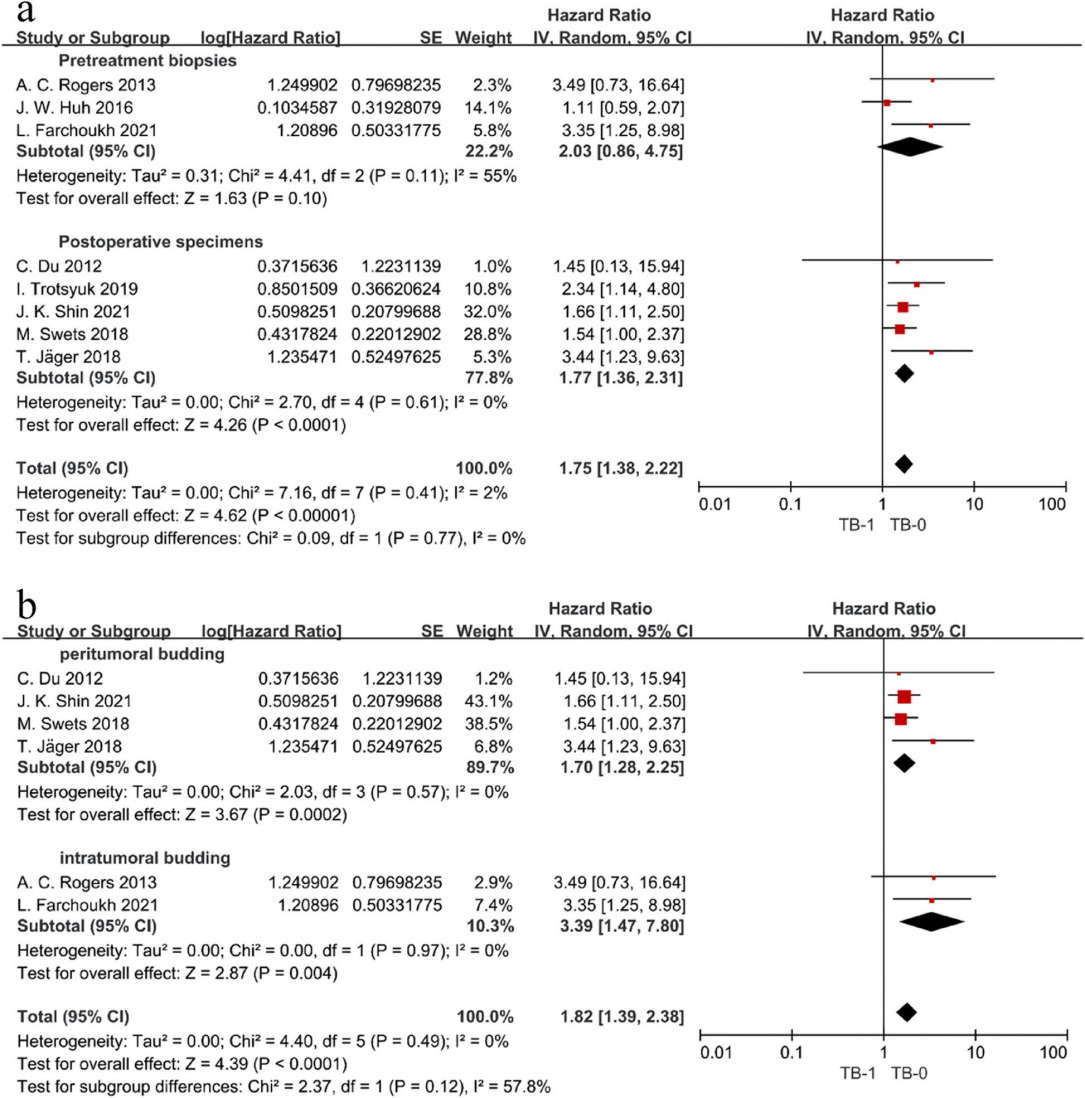
Forest plot of subgroup analysis comparing 5-year DFS between the TB-1 versus TB-0 groups. **a** Source of the specimen. **b** Location of tumor budding assessment

Data from the HR and 95% CI of the 5-year DFS multivariate regression analysis were provided in eight studies, and the meta-analysis showed no significant heterogeneity (*I*^2^ = 2%, HR = 1.75, 95% CI 1.38–2.22, *P* < 0.00001) (Fig. 2a). This indicated that the 5-year DFS was significantly lower in the TB-1 group than in the TB-0 group and that TB-1 was an independent poor predictor of 5-year DFS. Subgroup analysis revealed that the two subgroups of both pretreatment biopsies and postoperative specimens showed similar trends as described above (HR postoperative = 1.77, 95% CI 1.36–2.31, *P* < 0.0001), yet the pretreatment biopsy subgroup showed significant heterogeneity (*I*^2^ = 55%), with no statistically significant difference in the combined results (HR pretreatment = 2.03, 95% CI 0.86–4.75, *P* = 0.10) (Fig. 3a). After excluding the two included studies with unclear TB types, subgroup analysis showed that both PTB (HR = 1.70, 95% CI 1.28–2.25, *P* = 0.0002) and ITB (HR = 3.39, 95% CI 1.47–7.80, *P* = 0.004) increased the risk of adverse 5-year DFS (Fig. 3b). The Demir et al. study team analyzed the 1- and 3-year DFS rates comparing the two groups, which were 86% vs 93% and 24% vs 61%, respectively, and the results showed that the median DFS was significantly shorter in the TB-1 group than in the TB-0 group (HR = 3.14, 95% CI 1.42–6.94, *P* < 0.05) [26]. Five studies provided data related to 5-year OS, and meta-analysis of four of them showed *I*^2^ = 0%, HR = 1.77, 95% CI 1.21–2.59, *P* = 0.003, indicating that TB-1 was significantly associated with a poor 5-year OS (Fig. 2b).

##### LR and DM

Three studies provided both LR- and DM-related data, and the heterogeneity test revealed no significant heterogeneity (*I*^2^ = 0%). The pooled results showed that, statistically, TB-1 has significantly higher LR (OR = 4.15, 95% CI 1.47–11.75, *P* = 0.007) and DM rates (OR = 5.36, 95% CI 2.51–11.44, *P* < 0.0001) compared to TB-0 (Fig. 2c, d).

##### Other outcomes

Only two studies provided relevant data for the comparison of CSS between the two groups. The 5-year CSS rates reported in the studies were 73.8% vs 88.4% and 61.1% vs 87.3%, respectively (both *P* < 0.05), and the findings indicated that the 5-year CSS rates were lower in the TB-1 group than in the TB-0 group, with statistically significant differences (Table 3).

#### Correlation of pathological features

**Fig. 4:**
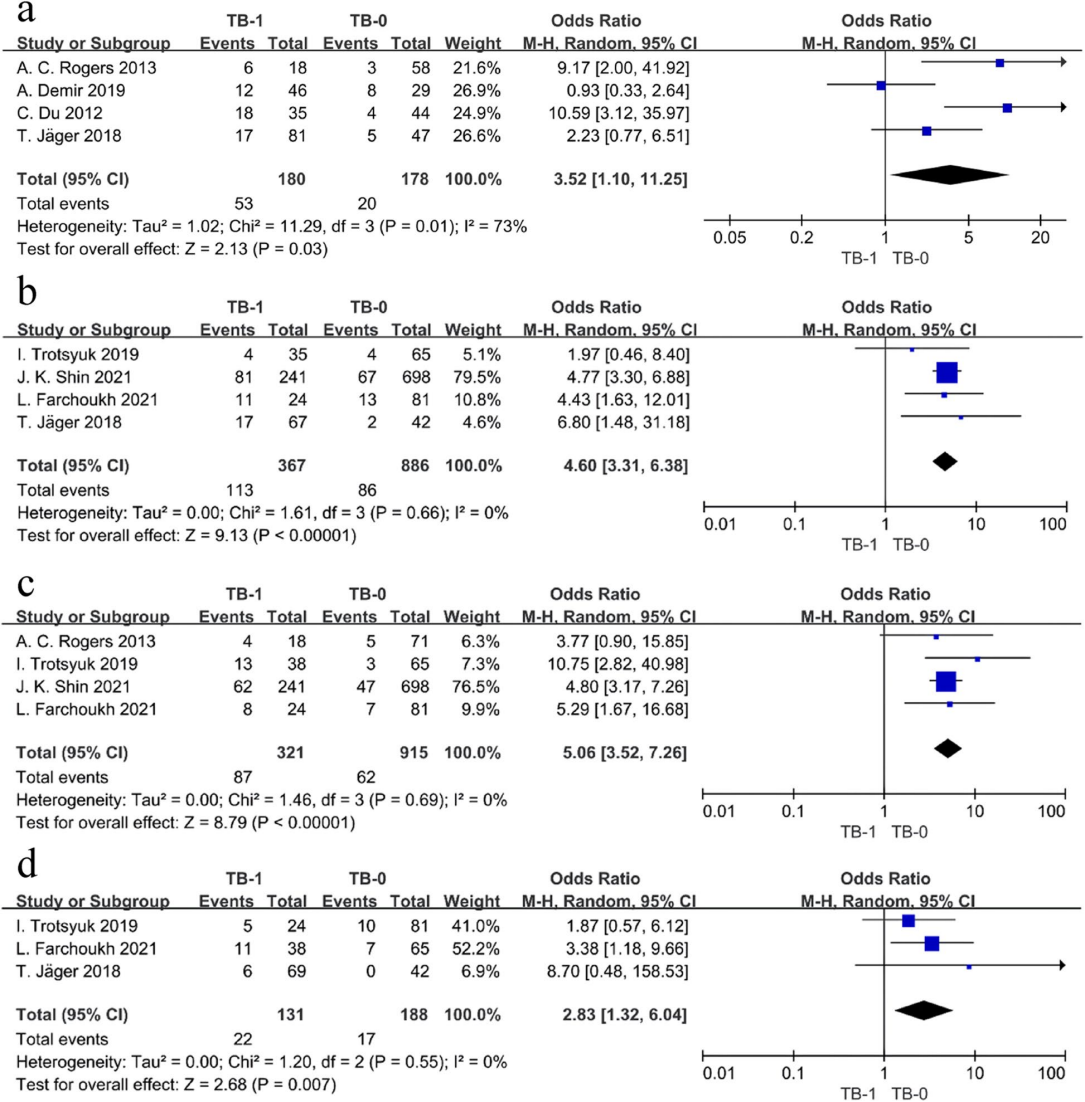
Forest plot comparing the correlation of pathological features between the TB-1 versus TB-0 groups. **a** Degree of differentiation. **b** Lymphatic invasion. **c** Perineural invasion. **d** Venous invasion

Four studies provided comparative information on the degree of differentiation between the two groups, and meta-analysis results showed a significant association between TB-1 and pathological presentation of lower differentiation (OR = 3.52, 95% CI 1.10–11.25, *P* = 0.03); however, there was a significant heterogeneity in the combination (*I*^2^ = 73%) (Fig. 4a). Four studies provided data on lymphatic invasion in both groups, and the pooled results showed no significant heterogeneity (*I*^2^ = 0%); TB-1 was significantly associated with the presence of lymphatic invasion by the tumor (OR = 4.60, 95% CI 3.31–6.38, *P* < 0.00001) (Fig. 4b). Four studies reported perineural invasion, and the combined results showed no significant heterogeneity (*I*^2^ = 0%); TB-1 was significantly associated with tumors presenting with perineural invasion (OR = 5.06, 95% CI 3.52–7.26, *P* < 0.00001) (Fig. 4c). Three studies reported venous invasion in both groups and the pooled results revealed no significant heterogeneity (*I*^2^ = 0%); a significant association was observed between TB-1 and tumors presenting with venous invasion (OR = 2.83, 95% CI 1.32–6.04, *P* = 0.007) (Fig. 4d).

#### Publication bias and sensitivity analysis

**Fig. 5:**
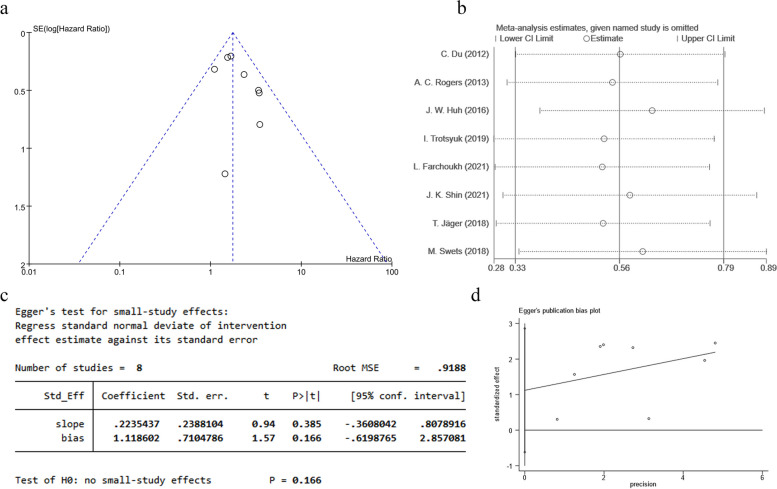
Publication bias and sensitivity analysis for 5-year DFS between the TB-1 versus TB-0 groups. **a** Funnel plot. **b** Sensitivity analysis plot. **c** Egger test results. **d** Egger test figure

The included studies with 5-year DFS were assessed for publication bias, and the results presented substantial symmetry on both sides of the funnel plot, with Egger test results showing *P* = 0.166 (*P* > 0.05) (Fig. 5a, c, d), indicating no significant publication bias. Sensitivity analysis of the 5-year DFS inclusion studies was performed using the one-by-one censoring method and revealed that the included studies were concentrated and within the 95% CI of the combined effect size, suggesting that the results of the meta-analysis were robust (Fig. 5b) (Additional file 3).

## Discussion

Currently, the management of colorectal cancer is mainly based on the TNM staging system and patients’ wishes for staging and stratification, and further adjuvant treatment is performed after assessing for the presence of high-risk factors. Nevertheless, the prognosis of patients at the same stage differs significantly, and researchers continue to explore prognosis-related factors to better guide precise clinical treatment. TB is an independent poor prognostic factor in colorectal cancer, and studies and meta-analyses have further validated that high-grade TB is significantly associated with lymph node metastasis in colorectal cancer (*P* < 0.05) [15, 30]. In addition, it was found that TB was significantly associated with poorer 5-year DFS, OS, and CSS in colorectal cancer (*P* < 0.001), and TB showed the same correlation with 5-year DFS, OS, and CSS in the neoadjuvant subgroup [16, 31]. However, the findings showed significant differences in the 5-year OS between rectal and colon cancers (*P* ≤ 0.001) [32, 33], and distinct differences in pathological manifestations, tumor immune microenvironments, and gene mutation profiles were found between tumor sites [21, 34]. The question therefore became: Does TB affect the prognosis of patients with rectal cancer following neoadjuvant therapy alone?

By pooling published studies, we found that TB-1 may be an independent predictor of lower 5-year DFS and OS and that TB-1 was significantly associated with high LR and DM rates. Notably, Jäger et al. performed a comparative analysis of the 5-year relapse-free survival rate in the TB-1 group, which we also performed in a meta-analysis because of its similar definition to that of DFS [21]. Six of the eight included studies compared the 5-year DFS rates between the two groups, and the pooled results showed a 5-year DFS of 33.3–71% in the TB-1 group and 75–90% in the TB-0 group (*P* < 0.05) (details are shown in Table 3), and the studies all concluded that TB-1 significantly affected the 5-year DFS rates in patients with rectal cancer undergoing neoadjuvant therapy. However, there was controversy among the included studies regarding whether TB-1 was a predictor of poor 5-year DFS. Five of the eight included studies concluded that TB-1 was an independent poor predictor of 5-year DFS compared to TB-0 (*P* < 0.05), which is similar to our findings [13, 18, 21, 23, 27]. In contrast, Huh et al. and Swets et al. performed multivariate regression analyses and found that TB-1 was not an independent poor predictor of 5-year DFS (*P* ≥ 0.05) [24, 28]. Furthermore, Huh et al. found that TB was not a predictor of 5-year DFS in a study after 3 years (*P* = 0.11 for univariate analysis) [22]. Huebner et al. showed that TB was significantly associated with 5-year DFS in a univariate regression analysis (*P* = 0.022); however, no multivariate analysis was performed to further explore its predictive value [25]. Demir et al. studied patients with rectal cancer who opted for neoadjuvant therapy with a median follow-up of 35 months, and multivariate regression analysis revealed that TB was an independent prognostic factor for DFS (*P* < 0.01) [26].

To clarify whether the source of specimens evaluated for TB differentially affects the 5-year DFS in patients with rectal cancer after neoadjuvant therapy, we performed a subgroup analysis and found that TB in both pretreatment biopsy and surgical resection was associated with a poorer 5-year DFS. The risk of poor 5-year DFS in patients with rectal cancer with TB-1 assessed in specimens appeared to be higher than that in pretreatment biopsies (HR pretreatment = 2.03 > HR postoperative = 1.75, Fig. 3a). However, it is possible that the few studies included in this subgroup resulted in no statistically significant difference in the pooled results, showing a need for further validation in the future. Additionally, we attempted to explore the impact of comparing PTB versus ITB on 5-year DFS, and after excluding two included studies with unknown definitions of TB, subgroup analysis revealed that both types of budding negatively affected 5-year DFS, and the risk of lower 5-year DFS may be higher for ITB than for PTB (HR-_PTB_ = 1.70 < HR-_ITB_ = 3.39, Fig. 3b). Farchoukh et al. found that ITB was significantly associated with the detection of TB-1 in surgically resected specimens after neoadjuvant therapy (*P* < 0.001), whereas Du et al. showed that significant necrosis and fibrosis of tumor glands after radiotherapy made the assessment of TB after neoadjuvant therapy more difficult, which may explain our findings [13, 18]. Notably, subgroup analysis is only a method of indirect inference, and results need to be verified by further studies.

Regarding the 5-year OS, a summary of four studies showed 5-year OS rates of 53–82.1% in the TB-1 group and 84–94.7% in the TB-0 group. Only the study by Jäger et al. shows no statistically significant difference in 5-year OS between the two groups (*P* = 0.09) (Table 3), which may be attributed to the fact that there were more cases in the TB-1 group than in the TB-0 group in that study, unlike other studies [13]. Whether TB-1 is a predictor of poor OS is equally controversial in previous studies. Some studies have shown that TB-1 is not an independent risk factor for OS [22, 28], while others have shown that TB-1 is a strong predictor of inferior OS after neoadjuvant therapy, even better than ypT and ypN status [23, 24, 27]. In addition, Demir et al. showed that TB-1 is a prognostic marker for poor DFS and OS in patients with rectal cancer, with or without neoadjuvant therapy [27].

Only three studies have analyzed LR and DM. Among them, Farchoukh et al. and Rogers et al. studied ITB, while Jäger et al. studied PTB, and their results suggested a greater likelihood of LR and DM (OR > 1) in the TB-1 group, which is similar to the results of our meta-analysis [13, 20, 21]. It is likely that the differences in the type of budding, neoadjuvant treatment modality, and postoperative adjuvant treatment caused the results of the Farchoukh et al. and Jäger et al. studies to show no statistical difference [13, 21]. PTB and ITB are significantly associated with reduced tumor T-stage downstaging and poorer pathological response to neoadjuvant therapy (*P* < 0.001) [13, 20, 21]. To the best of our knowledge, no study has compared the prognostic roles of the two budding types in patients after neoadjuvant therapy for rectal cancer. Recent studies have shown that patients with rectal cancer undergoing radical surgical resection after long-course nCRT still have a 1.2% likelihood of distal rectal mesenteric cancer cell spread, and distal surgical margins of 40 and 30 mm would result in 10% and 32% residual tumors, respectively [35]. Furthermore, compared to nCRT, patients with TNT and postoperative adjuvant treatment for rectal cancer receive longer cycles of systemic chemotherapy, and this treatment option is more likely to prevent and remove residual cancer cells and occult metastatic lesions in the area where the patient’s primary cancer is resected, thereby diminishing the likelihood of LR and DM. However, systematic reviews and meta-analyses found that, regardless of high risk, patients with stage III rectal cancer who underwent radical resection after nCRT did not benefit from adjuvant chemotherapy [36]; the differences between the TNT and nCRT groups with respect to LR and DM were not statistically significant (LR: OR = 1.82, 95% CI 0.95–3.49, *P* = 0.07; DM: OR = 0.77, 95% CI 0.58–1.03, *P* = 0.08) [37].

In contrast to analyzing TB alone, Swets et al. analyzed TB separately and in combination with other adverse pathological features such as lymphatic, perineural, extramural venous, and intramural venous invasion. This study found that TB-1, perineural invasion, and extramural venous invasion were all associated with reduced OS and DFS in patients with rectal cancer undergoing radical resection after short neoadjuvant radiotherapy, that patients with ≥ 2 adverse pathologic features had a higher risk of adverse OS and DFS and DM after neoadjuvant treatment, and that neither alone nor in combination with adverse pathologic features was found to be effective in predicting the benefit of postoperative adjuvant chemotherapy [24]. Kim et al. analyzed TB in combination with lymphatic invasion, perineural invasion, and venous invasion as four risk factors and found that two factors (medium-risk group) and more than three factors (high-risk group) were poor independent predictors of 5-year DFS and OS after radical resection following neoadjuvant therapy in patients with rectal cancer (*P* < 0.001). Moreover, postoperative adjuvant chemotherapy was a prognostic factor associated with 5-year OS in patients with rectal cancer who underwent neoadjuvant therapy in the low-medium–high risk group (≥ 1 factor) (*P* ≤ 0.007) [29]. Shivji et al. combined tumor outgrowth and hypofractionated clusters into a “combined score” (CS) to predict the prognosis of stage I–III colorectal cancer, and multifactorial analysis found that high-grade CS was significantly associated with poorer DFS, OS, and CSS, respectively (*P* = 0.0002, 0.009, and 0.005); however, it was not controlled for tumor type and neoadjuvant treatment factors [16]. Therefore, we attempted to study the correlation between tumor budding and other adverse pathological features and found that TB-1 was significantly associated with adverse pathological features such as poor differentiation and lymphatic, perineural, and venous invasion (*P* < 0.05); however, the predictive role of TB combined with and without other adverse pathological features on the prognosis of patients with rectal cancer after neoadjuvant therapy needs to be further researched.

## Limitations

To our knowledge, ours is the first systematic review and meta-analysis on the impact of TB on the prognosis of patients with rectal cancer after neoadjuvant therapy. However, some limitations exist in our study: (1) The included studies were all retrospective, and there is an inherent effect of bias in such studies. (2) Inconsistent criteria for TB grouping may have reduced the reliability of the study results. The TB grouping criteria remain controversial. Although the ITBCC strongly recommends a three-tier system for low-, intermediate-, and high-grade TB, most studies ultimately choose to divide the study into low- and high-grade TB groups, given the sample size and convenience of statistical analysis [11]. (3) Differences in neoadjuvant and postoperative adjuvant therapy regimens among the included studies may have caused inconsistencies in the study results and realistic clinical outcomes.

## Conclusion

We found a significant association between tumor budding and adverse prognosis as well as poor pathological features following neoadjuvant therapy for rectal cancer. Identifying the level of tumor budding can assist in selecting an appropriate treatment regimen requiring further investigation for patients with rectal cancer after neoadjuvant therapy. It is anticipated that future high-quality, randomized, controlled trials will be conducted to validate our findings.

### Supplementary Information

**Additional file 1.** Detailed search strategy.

**Additional file 2.** The NOS score table of the included literature.

**Additional file 3.** Sensitivity analyses for combining outcomes and pathologic features with significant heterogeneity by the one-by-one exclusion method.

## Data Availability

Data were extracted from published sources.

## Abbreviations

CI: Confidence interval
CSS: Cancer-specific survival
DFS: Disease-free survival
DM: Distant metastasis
HR: Hazard ratio
ITB: Intratumoral budding
TNM: Tumor-node-metastasis
ITBCC: International Tumor Budding Consensus Conference
LR: Local recurrence
nCRT: Neoadjuvant chemoradiotherapy
NOS: Newcastle Scale
OR: Odds ratio
OS: Overall survival
PTB: Peritumoral budding
TB: Tumor budding
TB-0: Low-grade or negative tumor budding
TB-1: High-grade or positive tumor budding
TNT: Total neoadjuvant chemoradiotherapy
