# Supplementary material for: Prognostic impact of tumor budding in rectal cancer after neoadjuvant therapy: a systematic review and meta-analysis
Source: Syst Rev. 2024 Jan 9;13:22. doi: 10.1186/s13643-023-02441-9 (PMC10775462; doi:10.1186/s13643-023-02441-9)
Supplement: Supplementary file 1 — Additional file 1. Detailed search strategy. [file 13643_2023_2441_MOESM1_ESM.doc]

Search strategy

Pubmed

(((((((((((((((((Neoplasm, Rectal[Title/Abstract]) OR (Rectal Neoplasm[Title/Abstract])) OR (Rectum Neoplasms[Title/Abstract])) OR (Neoplasm, Rectum[Title/Abstract])) OR (Rectum Neoplasm[Title/Abstract])) OR (Rectal Tumors[Title/Abstract])) OR (Rectal Tumor[Title/Abstract])) OR (Tumor, Rectal[Title/Abstract])) OR (Neoplasms, Rectal[Title/Abstract])) OR (Cancer of Rectum[Title/Abstract])) OR (Rectum Cancers[Title/Abstract])) OR (Rectal Cancer[Title/Abstract])) OR (Cancer, Rectal[Title/Abstract])) OR (Rectal Cancers[Title/Abstract])) OR (Rectum Cancer[Title/Abstract])) OR (Cancer, Rectum[Title/Abstract])) OR (Cancer of the Rectum[Title/Abstract])) AND (((((Tumor budding[Title/Abstract]) OR (Tumour budding[Title/Abstract])) OR (Budding of tumor[Title/Abstract])) OR (Budding of tumour[Title/Abstract])) OR (Budding[Title/Abstract])) 80

Time 2023-1

Embase

#1 'neoplasm, rectal' OR 'rectal neoplasm'/exp OR 'rectal neoplasm' OR 'rectum neoplasms'/exp OR 'rectum neoplasms' OR 'neoplasm, rectum' OR 'rectum neoplasm'/exp OR 'rectum neoplasm' OR 'rectal tumors' OR 'rectal tumor'/exp OR 'rectal tumor' OR 'tumor, rectal' OR 'neoplasms, rectal' OR 'cancer of rectum' OR 'rectum cancers' OR 'rectal cancer'/exp OR 'rectal cancer' OR 'cancer, rectal' OR 'rectal cancers' OR 'rectum cancer'/exp OR 'rectum cancer' OR 'cancer, rectum'/exp OR 'cancer, rectum' OR 'cancer of the rectum':ab,ti 88780

#2 'tumor budding' OR 'tumour budding' OR 'budding of tumor' OR 'budding of tumour' OR 'budding':ab,ti 26415

#1 and #2 216

Cochrane library 10

Clinicalkey 5

CNKI(China National Knowledge Infrastructure)

Keywords or abstracts:（rectal cancer or rectal carcinoma） and Tumor budding 56

Wanfang database：

Title or Keywords: (rectal cancer) and (Tumor budding) 57
