# Supplementary material for: Prognostic impact of tumor budding in rectal cancer after neoadjuvant therapy: a systematic review and meta-analysis
Source: Syst Rev. 2024 Jan 9;13:22. doi: 10.1186/s13643-023-02441-9 (PMC10775462; doi:10.1186/s13643-023-02441-9)
Supplement: Supplementary file 2 — Additional file 2. The NOS score table of the included literature. [file 13643_2023_2441_MOESM2_ESM.doc]

|  |  | Score of NOS Assessment | | |  | type of study |  |  |
| --- | --- | --- | --- | --- | --- | --- | --- | --- |
| author | years of issue | selection | comparability | outcome/exposure |  |  |  |  |
| C. Du | 2012 | *** |  | ** | 5 | retrospective case-control study |  |  |
| M. Huebner | 2012 | **** |  | *** | 7 | retrospective cohort study |  |  |
| A. C. Rogers | 2013 | **** |  | *** | 7 | retrospective cohort study |  |  |
| J. W. Huh | 2016 | **** |  | ** | 6 | retrospective cohort study |  |  |
| T. Jäger | 2018 | **** |  | *** | 7 | retrospective cohort study |  |  |
| M. Swets | 2018 | **** |  | *** | 7 | retrospective cohort study |  |  |
| A. Demir | 2019 | **** |  | ** | 6 | retrospective cohort study |  |  |
| J. W. Huh | 2019 | **** |  | ** | 6 | retrospective cohort study |  |  |
| I. Trotsyuk, H | 2019 | **** |  | *** | 7 | retrospective cohort study |  |  |
| L. Farchoukh | 2021 | **** |  | *** | 7 | retrospective cohort study |  |  |
| J. K. Shin | 2021 | **** | ** | * | 7 | retrospective cohort study |  |  |

Score of NOS Assessment
