# Supplementary material for: Prognostic impact of tumor budding in rectal cancer after neoadjuvant therapy: a systematic review and meta-analysis
Source: Syst Rev. 2024 Jan 9;13:22. doi: 10.1186/s13643-023-02441-9 (PMC10775462; doi:10.1186/s13643-023-02441-9)
Supplement: Supplementary file 3 — Additional file 3. Sensitivity analyses for combining outcomes and pathologic features with significant heterogeneity by the one-by-one exclusion method. [file 13643_2023_2441_MOESM3_ESM.docx]

**Supplementary file 3: Sensitivity analyses for combining outcomes and pathologic features with significant heterogeneity by the one-by-one exclusion method were performed as follows:**

**Fig. 3a: Subgroup analysis by source of specimen comparing 5-year DFS between TB-1 versus TB-0 groups**

| Exclusion study | I2 | HR | P |
| --- | --- | --- | --- |
| A. C. Rogers 2013 | 71% | 1.80 [0.61, 5.27] | 0.28 |
| J. W. Huh 2016 | 0% | 3.39 [1.47, 7.80] | 0.004 |
| L. Farchoukh 2021 | 44% | 1.56 [0.56, 4.35] | 0.40 |
| Unexcluded | 55% | 2.03 [0.86, 4.75] | 0.10 |

**Interpretation:** The combined results after study-by-study exclusion showed a significant reduction in I2 after excluding the J. W. Huh 2016 study, P<0.05, considering that the heterogeneity of the meta-analysis of this outcome may have originated from this study, and that the combination of the results was not robust possibly due to the small number of included studies, and that the results need to be interpreted with caution.

**Fig. 4a: Correlation of degree of differentiation between TB-1 versus TB-0 groups**

| Exclusion study | I2 | OR | P |
| --- | --- | --- | --- |
| A. C. Rogers 2013 | 77% | 2.71 [0.70, 10.47] | 0.15 |
| A. Demir 2019 | 53% | 5.60 [1.95, 16.13] | 0.001 |
| C. Du 2012 | 66% | 2.40 [0.73, 7.89] | 0.15 |
| T. Jäger 2018 | 82% | 4.27 [0.79, 23.01] | 0.09 |
| Unexcluded | 73% | 3.52 [1.10, 11.25] | 0.03 |

**Interpretation:** The combined results after excluding studies one by one showed no significant reduction in I2 but a significant change in P value, the source of heterogeneity could not yet be determined by sensitivity, and the combined results were not robust possibly due to the small number of included studies, and the results need to be interpreted with caution.
